# The Emerging Role of The Eosinophil and Its Measurement in Chronic Cough

**DOI:** 10.2174/1874306401711010017

**Published:** 2017-06-30

**Authors:** Mahboobeh H. Sadeghi, Alyn H. Morice

**Affiliations:** Respiratory Medicine, Castle Hill Hospital, Centre for Cardiovascular and Metabolic Research, Hull York Medical School, Cottingham, UK.

**Keywords:** Chronic Cough, Eosinophil, Cough variant asthma, Eosinophilic bronchitis, Montelukast, Innate immunity

## Abstract

Although the aetiology of chronic cough in guidelines is clearly stated as asthma and related syndromes, gastro-oesophageal reflux disease (GORD), and upper airways disease, the inflammatory mechanisms underlying these conditions differ. Recent studies on asthma have increasingly focused on its molecular phenotypes instead of clinical characteristics. Here, we proposed the hypothesis that divides cough into two groups; the eosinophilic and neutrophilic. This division will enhance our ability to recognise the type of airway inflammation which, as a consequence will lead us to more targeted and personalized treatment approaches.

## INTRODUCTION

Cough is one of the most common symptoms in respiratory disease which forces patients to seek medical attention. The etiological mechanisms of cough is poorly understood causing a challenge to the management [1]. Even after a clear diagnosis, it is still difficult to control and manage cough, and this decreases the quality of life in cough patients [2].

For the majority of patients who suffer from acute cough the cause is usually a viral respiratory tract infection. This is initially disruptive but is generally self-limiting [3]. Conversely, chronic cough is a continual symptom which is caused by many chronic respiratory, upper airway and gastrointestinal disease [4]. Many patients report that their chronic cough was preceded by an acute respiratory tract infection suggesting a common mechanism of cough hypersensitivity.

## CHRONIC COUGH

Chronic cough is a common but poorly diagnosed condition. It has been typically defined as a cough that persists for more than eight weeks [5]. Epidemiological surveys reveal that approximately 10% of the population suffer from a chronic cough [6, 7].

Chronic cough has a significant impact on physical and psychological morbidity. Patients suffer from various physical symptoms including chest pain, headaches, sore throat, voice changes, vomiting, incontinence, sleep deprivation and syncope [6, 8, 9]. Chronic cough also has a negative impact on patients’ relationships and social life and can lead to social isolation. Thus, anxiety and depression is common among these patients [6, 10, 11]. An understanding of the pathogenesis of chronic cough is vital to design more effective treatment.

## AETIOLOGY OF CHRONIC COUGH

In the literature it has been reported that there are three common aetiologies of chronic cough: 1)asthma and related syndromes, 2) gastro-oesophageal reflux disease (GORD), and 3) upper airways cough syndrome characterised by postnasal drip, rhinitis, and sinonasal disease. However, despite following diagnostic algorithms in cough guidelines, in many patients the cause of cough remains obscure leading to term “idiopathic” chronic cough [5, 7, 12, 13]. To overcome this diagnostic uncertainty an overarching syndrome based on the observed hypersensitivity to external noxious stimuli was proposed. Evidence for this approach was obtained in a worldwide survey of 10032 chronic cough patients from 11 cough clinics. Patients appeared to have a single, common, clinical entity [14].This survey has shown that there is a common clinical history and demographic profile in these patients. We have suggested that the Cough Hypersensitivity Syndrome is a consequence of a form of gastro-oesophageal disease [15]. We hypothesize that reflux causes inflammation in the airways which in some leads to an eosinophilic response thus giving rise to asthma like syndromes. This airway reflux also irritates the nasal passages and sinuses giving rise to upper airways disease [16]. Differences in the prevalence of chronic cough phenotypes may depend on access to specialists, their particular interest and in their understanding of the causes of cough [12, 17]. Here, we suggest that different phenotypes of chronic cough are due to differences in the profile of inflammation seen in individual patients and hypothesise that important therapeutic consequences are dependent on these difference.

### 1 Asthma And Related Syndromes

Chronic cough is one of the predominant asthma symptoms. However, what defines the diagnosis of “asthma” is unclear [18]. It has been claimed that there are three different asthmatic conditions which lead to isolated chronic cough: 1) cough variant asthma, 2) atopic cough, and 3) eosinophilic bronchitis.

Patients with cough variant asthma characteristically suffer from non-productive cough in the absence of other asthma symptoms such as wheezing and dyspnea [19]. This cough is characterised with bronchial hyper responsiveness and eosinophilia in both sputum and broncho alveolar lavage (BAL), but without bronchoconstriction or airway obstruction [12, 19, 20].

Atopic cough is characterised by cough hypersensitivity and sputum eosinophilia in the absence of bronchial hyper responsiveness and airway obstruction [21]. A history of an atopic constitution may be found in this group of patients [12, 20]. Whether these patients truly represent a separate subgroup has been challenged [22].

Eosinophilic bronchitis is a condition in which chronic cough is present with sputum eosinophilia without variable airflow obstruction (bronchoconstriction) and bronchial hyperresponsiveness [23]. Eosinophilic bronchitis chronic cough may be refractory to inhaled anti-asthma therapy but they respond to high doses of parenteral steroids [24].

### 2 Gastro-Oesophageal Reflux Disease

Classic gastro-oesophageal reflux disease (GORD) has been identified in 2 - 40% of cases in prospective studies of chronic cough. However, diagnosis of the more important extra-oesophageal reflux is problematic as there are no clear diagnostic criteria [25].

Classically, the retrograde movement of acid and other gastric contents of the stomach into the oesophagus and beyond is termed gastro-oesophageal reflux. In the majority of patients, this is due to impaired function or brief relaxation of the lower oesophageal sphincter (LOS). GORD causes symptoms such as acid regurgitation, and heart burn which improve by anti-acid therapy such as the proton pump inhibitors (PPIs) [5, 13]. However, extra-oesophageal non-acid reflux may be an aerosol and gaseous. We have suggested that “The reflux which causes respiratory consequences is a gaseous mist which is partially or even wholly non-acid. This mist can travel up the oesophagus without a peristaltic wave since the oesophagus, as is usually seen on thoracic computed tomography, is patent; a so-called common cavity” [16]. Normally, the LOS opens and allows this gas to pass. However, when this gaseous mist excessively increases in the airway or in some cases if the airway becomes sensitive, it can cause inflammation in the respiratory tract, as well as the nose, ears and sinuses [16]. The symptom profile arising from is termed“ airway reflux” as we have called it. Other terms for the same phenomenon are extra esophageal reflux, laryngopharyngeal reflux (LPR), and silent reflux. The latter because heartburn and regurgitation which are typical symptoms of GORD may be absent [26]. Objective evidence of airway reflux may be obtained by impedance measurements or the detection of pepsin in saliva [27]. However such techniques are imprecise and the clinical history is in our opinion key to the diagnosis. This symptom complex has been codified in a validated questionnaire which is called Hull Airways Reflux Questionnaire(HARQ). This validated questionnaire supports the diagnosis of extra oesophageal reflux associated with cough in the majority of patients with chronic cough [28, 29].

## 3 Upper Airways Disease

Postnasal drip (PND) is defined as a sense of nasal secretions or dripping into the back of the throat from the nose or sinuses. PND is said to lead to symptoms such as cough, frequently clearing the throat (throat-clearing) and nasal discharge or nasal stuffiness. Belief in the syndrome arose from the observation of first generation of antihistamines causing an improvement in cough in significant number of patients [30]. However, this is a central effect, since potent second generation of antihistamines which do not pass the blood brain barrier have no effect on chronic cough [31] Gaseous airway reflux may cause upper airways symptoms by epithelial damage in the upper respiratory tract and irritation of the larynx and cough receptors [32, 33].

## COUGH INFLAMMATION PHENOTYPES

Recently, molecular phenotypes of asthma (eosinophilic asthma and neutrophilic asthma) based on inflammatory biomarkers have been developed to understand the mechanistic insights and pathology of asthma [34]. Similarly in cough, it has been evident that cough variant asthma, atopic cough, and eosinophilic bronchitis (EB) present an eosinophilic phenotype in cough patients [12, 20, 21, 23, 35]. Studies from secondary care indicate that about 20% of patients with chronic cough have eosinophilic inflammation present in their airway, whereas the remaining patients tend to have a neutrophilic phenotype [36]. This is important because those patients with eosinophilic inflammation respond to anti-inflammatory therapy [36]. Treatment of the EB can be highly effective, but classic asthma treatment often does not lead to complete resolution. Because the element of bronchoconstriction is missing in ‘asthmatic cough’ bronchodilators are not effective. The inflammation in asthmatic cough may also be more deep seated [37]. Asthmatic cough is a well-recognised phenomenon by physicians and so most patients with a chronic cough receive a trial of anti-asthma medication such as inhaled or oral corticosteroids. However, it is not the only phenotype of cough, the majority having chronic non-specific inflammation. These patients do not respond and so dose escalation is common. Indeed, many patients are given potentially harmful therapy such as parenteral steroids with little chance of therapeutic response.

## IMPORTANCE OF INNATE IMMUNE SYSTEM IN EOSINOPHILIC INFLAMMATION

Eosinophilic airway inflammation characterised in sputum, bronchoalveolar lavage (BAL) fluid and bronchial biopsies. Classically, it is believed T helper 2 cells (Th2) by releasing interleukin 4 (IL-4), IL-5, and IL-13 play a vital role to induce eosinophilic allergic asthma. Therefore, new treatment of asthma has been developed which blocks these cytokines [38]. Recently, the role of the innate immune system rather than the adaptive immune system in eosinophilic non-allergic asthma has been highlighted. Members of the innate lymphoid family, ILC group 2 produce type2 cytokines which are suggested to play an important part in the genesis of airway eosinophilic inflammation [38-42].

## T HELPER 2 CELLS AND EOSINOPHILIA

The adaptive immune system has long been recognised as the major factor in allergic asthma through epithelial cell-derived cytokines TSLP, IL-25 and IL-33 secreted during allergen exposure. As a result, Th2 cells release large amounts of inflammatory cytokines (IL-4, IL-5, and IL-13) [43, 44].

IL-4 stimulates B cells class switching to IgE, which can bind to high-affinity IgE receptors (FcεR1) on basophils and mast cells. Activated basophils and mast cells release inflammatory mediators such as cytokines, chemokines, histamine, heparin, serotonin and proteases. These mediators cause constriction in smooth muscles, increase vascular permeability and mucus hypersecretion [43, 45, 46]. IL-5 induces bone marrow to produce eosinophils leading to tissue eosinophilia and airway remodelling [39, 45, 46]. IL-13 mediates to increase production of mucus in airway epithelia and smooth muscle cells, and is necessary to promote airway hyperresponsiveness (AHR) [43, 45, 46].

## ILC2 CELLS AND EOSINOPHILIA

Recently, the involvement of the innate immune system in airway eosinophilic inflammation has been clarified through a series of studies on humans and mice. Anti-IL-4 and anti-IL5 therapies in humans led to a favourable result in asthmatic patients with a high level of eosinophils. Surprisingly, these patients responded to the therapies regardless of being atopic or not [39]. Non atopic administration of IL-25 in mice induces the production of IL-4, IL-5, IL-13, IgE, IgG1 and IgA. Airway eosinophilia was then produced by IL25 in RAG-deficient mice, which do not have B cells or T cells [42]. Administration of IL-25 and co-infection of mice with Nippostrongylus brasiliensis [47] and helminth [48] in other studies, revealed similar results. These findings indicated that type2 cytokines and eosinophilia can be produced without the adaptive immune system activation. Therefore, the recent discovery that innate lymphoid cells (ILC) are another important source of T cell-associated cytokines has important consequences for our understanding of airway inflammation in EB.

ILCs are activated by IL-33 or a combination of IL-2 and IL-25, and they express Sca-1, c-Kit, IL-33R, and IL-7R. However, they do not express antigen-specific receptors or lineage markers (CD3, CD4, CD8, TCR, TCR, CD5, CD19, B220, NK1.1, TER119, Gr-1, Mac-1, CD11 and FceRIa) thus, they cause nonspecific immune responses. Innate lymphoid cells are classified into three groups based on their ability to secrete Th cell-associated cytokines. ILC1 secrete interferon-γ (IFN-γ), ILC2 produce IL-5 and IL13 and finally ILC3 produce IL-17 and IL-22 [38, 42, 43, 48, 49].

ILC2 are activated in the presence of IL-25, IL-33 or thymic stromal lymphopoietin (TSLP). They are dependent on the TH2-defining transcription factor GATA-binding protein 3 (GATA-3) and the transcription factor retinoic acid receptor-related orphan receptor (RORa) for their development. GATA-3 is required to produce ILC lineages in bone marrow for ILC2 differentiation and maintenance [50] Fig. (**1**).There is thus a clear molecular mechanism for the production of non-atopic EB in patients with chronic cough. Epithelial damage induces the innate immune system to release TH2 cytokines without the need for an adaptive response.

## BIOMARKERS OF AIRWAY INFLAMMATION; DIAGNOSTIC APPROACHES

In eosinophilic inflammation, the numbers of eosinophil cells in peripheral blood and in airways secretions are increased. Several studies showed that the number of eosinophil cells have a correlation with the severity of asthma. For example, Bousquet and colleagues [51] have reported that the number of eosinophils in peripheral blood and in bronchial lavage of asthmatic patients is associated with severity of asthma. Brightling and colleagues [36] also showed that chronic cough patients with EB had higher numbers of eosinophil in their sputum which reduced significantly after inhaled corticosteroid therapy. Therefore, the measurement of airway eosinophilia is an important biomarker to correctly assess the inflammation type and severity. Consequently, it helps to employ therapies which target airway inflammation specifically.

Bronchoalveolar lavage and endobronchial biopsies are the gold standard methods to detect eosinophilic inflammation. However, these methods are invasive and costly and also have many other disadvantages. Therefore, a number of non-invasive sampling methods have been developed which are reliable and complementary to the reference standards. In this section we will discuss sputum induction, blood eosinophilic biomarkers and measurement of fractional exhaled nitric oxide (FeNO) methods as diagnostic approaches to the assessment of eosinophilic inflammation [52, 53].

### 1 Sputum Induction

According to the European Respiratory Society (ERS) guidelines sputum induction is a validated tool to diagnose respiratory inflammation and monitor anti-inflammatory drug outcomes [54]. Sputum induction is performed to collect an adequate sample of secretions from lower airways. In this procedure inhalation of hypertonic saline solution by nebulisation helps the subject to produce sputum that can be expectorated [55].

Sputum induction in comparison with bronchial biopsy or BAL biopsy is safer, cheaper and easier to administer. However, there are some limitations that reduce success rate of this technic. Inhalation of hypertonic saline is an unpleasant experience for subjects which may cause bronchoconstriction. Not all patients are able to produce sputum, even if they produce a sample not all the samples are suitable for analysis. Analysing the sputum sample is a time-consuming and difficult procedure which requires adequate equipment and a highly trained technician [52].

## 2 Blood Eosinophil Count 

Measurement of blood eosinophil count is another valuable, easy and non-invasive method to identify patients with EB. Blood eosinophil count measures systemic eosinophilic inflammation that is an indirect but useful tool to assess airways inflammation severity, and consequently predict and direct treatment in respiratory patients [56, 57].

Several studies show that there is a positive relationship between sputum eosinophils and blood eosinophils. However, there is only a moderate correlation between these two components [56-58]. It is believed that the systemic inflammation plays an independent role in asthma and other respiratory diseases [59]. Biologically eosinophils are produced in bone marrow consequent to secretion of inflammatory cytokines such as IL-5. Eosinophils are released into the blood and transported to the target tissue [60]. Thus, IL-5 may increase the eosinophilia in both blood and sputum. It is suggested that combining the evaluation both local and systemic eosinophilic inflammation in respiratory disease may provide complementary data of greater value [56, 58, 61].

## 3 Measurement Of Fractional Exhaled Nitric Oxide (Feno)

The measurement of exhaled nitric oxide is widely accepted as a non-invasive marker of airway Inflammation and, amongst other uses, has been proposed to monitor the response to anti-inflammatory medications. In 2005 clinical guidelines for the measurements of nitric oxide (NO) from the upper and lower respiratory tract has been published by the American Thoracic Society (ATS) [52].

Previous studies have shown that a rise in FeNO value can be detected in patients with asthma and a further increase has been seen during exacerbations [52]. Biologically the level of nitric oxide in breath is associated with secretion of IL-4 and IL-13 [58]. Thus there is a correlation between FeNO concentrations and the IgE levels and the positive skin prick test [62]. Likewise, patients with atopic asthma produce higher levels of FeNO than patients with non-atopic asthma [63].

In a recent study it has been found that the prevalence of current asthma and wheeze increased 3 times more among patients with high FeNO values than patients with normal FeNO [58]. Moreover, FeNO level is decreased in response to anti-inflammatory treatments. It had been shown that the level of FeNO decreases with anti–IL-13 treatment (lebrikizumab) [64]. In response to inhaled corticosteroids FeNO level reduction was dependent on to the dose of treatments [65]. Administration of leukotriene receptor antagonists also resulted in reduction in FeNO level [66].

It has been reported that the level of FeNO in patients using corticosteroids decreases quickly while airway inflammation and hyperresponsiveness can still be detected by other markers of airway inflammation [67]. Therefore, the reliability of FeNO as a guide to therapy could be questioned and FeNO might be too sensitive to the initiation of corticosteroid therapy.

## BLOOD EOSINOPHIL COUNT OR FeNO

FeNO and blood eosinophilia have been considered as replacement markers for sputum eosinophilia as each of them is able to distinguish eosinophilic inflammation phenotype from neutrophilic. However there is only a modest correlation between FeNO and sputum eosinophilia (r=0.59, p<0.001), and a moderate to good correlation between blood and sputum eosinophilia (r=0.52, p<0.001) in asthmatic patients [56]. A systematic review and meta-analysis study in asthma reported that receiver operating characteristics area under the curve (ROC AUC) for FeNO in 17 adult studies (3216 patients) was 0·75 (95% CI 0·72–0·78). The ROC AUC for blood eosinophilia in 14 adult studies (2405 patients) was 0·78 (0·74–0·82) [53]. Accordingly, blood eosinophilia and FeNO appeared to be the best predictor for eosinophilic inflammation, though their diagnostic accuracy consistently reflected moderate association [53, 56]. However, it is important to consider that studies on asthmatic patients might not reflect results in chronic cough patients. In a systematic review that investigated the diagnostic accuracy of FeNO in chronic cough patients in 15 studies has shown that AUC for FeNO on patients with cough variant asthma was 0.87 (95% CI 0.84-0.90). The diagnostic accuracy of FeNO on chronic cough patients with cough variant asthma or eosinophilic bronchitis was AUC 0.89 (95% CI 0.86-0.92) while it was AUC=0.81 (0.77-0.84) on chronic cough patients with non-asthmatic eosinophilic bronchitis [68].

Consequently, based on the above evidence, it is believed that these biomarkers cannot individually diagnose eosinophilic inflammation accurately. Particularly, it is evident that FeNO and blood eosinophilia are triggered by two different cytokine mechanisms and there is a weak correlation between these two elements [56, 58]. As a result, it is suggested that a combination of these markers with other clinical features is more preferable, which is expected to improve diagnostic accuracy [52, 53, 58]. This however needs to be tested against clinical endpoints, particularly in eosinophilic cough.

## AIRWAY INFLAMMATION; TREATMENT APPROACHES

Despite substantial clinical investigation on anti-cytokines therapy, treating airway inflammation has, as yet, seen partial success [46]. Corticosteroid therapy remains the most widespread anti-inflammatory treatment, yet side effects related to this therapy have raised concerns, particularly when the majority of patients with neutrophilic inflammation respond poorly or not at all to high-dose inhaled or oral steroid therapy [25].

Montelukast is another treatment preference that is generally considered as an add-on therapy in patients with poorly controlled asthma. However, recently it has been suggested that montelukast might have wider range of anti-inflammatory properties than originally thought [69].

## CORTICOSTEROIDS THERAPY

Corticosteroids have been supported as an anti - inflammatory therapy in airway diseases for five decades. Systemic corticosteroids (SCS) and inhaled corticosteroids (ICS) are two forms of steroid therapy which have been used in airway inflammation.

ICSs are widely used in chronic cough [70]. ICSs are effective on eosinophilic bronchitis where they generally improve cough and treat airway eosinophilia [36]. ICSs are also useful for patients with cough variant asthma (CVA) and in the long term it has been suggested that it may help to prevent progression to classic asthma [71]. Conversely, SCSs are infrequently used to control eosinophilic bronchitis [37]. According to the American College of Chest Physicians (ACCP) Evidence-Based Clinical Practice Guidelines [72]“Patients with cough due to asthma should initially be treated with a standard antiasthmatic regimen of inhaled bronchodilators and inhaled corticosteroids (ICSs). In patients whose cough is refractory to treatment with ICSs, an assessment of airway inflammation should be performed whenever available and feasible. The demonstration of persistent airway eosinophilia during such an assessment will identify those patients who may benefit from more aggressive anti-inflammatory therapy” [72].

## CLINICAL STUDIES REVIEW OF ORAL PREDNISOLONE

Whilst oral prednisolone is a SCS which is the most commonly used steroid in the treatment of patients with chronic asthma [73] in chronic cough, there are no studies that assess the effects of oral prednisolone apart from some small trials which have shown its effects on patients with cough variant asthma (CVA). In a small prospective open label study oral prednisone was used to diagnose CVA in a group of patients who suffered from persistent cough from 2 months to 20 years. This diagnostic - therapeutic trial revealed that nine out of 10 patients responded to a short course of oral prednisone, and then treatment followed by corticosteroid inhalers to control cough and maintain the result [74]. Similar to this study Cheriyan and colleagues (1994) characterised CVA as a persistent nonproductive cough with minimal wheezing or dyspnea. They reported similar results on a small study among 10 patients [71]. However none of these studies characterised the type of inflammation associated with cough.

In addition to the lack of literature and large randomised control trials in this area, there is no consistent guidelines on dose or duration of corticosteroids therapy for treatment of cough syndromes [70].

By considering all the present evidence of prednisolone efficacy on airway inflammation and its many unwanted effects, particularly in the chronic therapy, we believed that it is necessary to fully evaluate the relative efficacy of prednisolone and montelukast in chronic cough. Brightling (2006) believed that “The role of other potential therapeutic agents such as antihistamines and antileukotrienes needs to be fully explored” [37].

## MONTELUKAST

Montelukast is a pharmacological antagonist of type 1 cysteinyl leukotriene receptors (CysLT1Rs). Cysteinyl leukotriene (cysLTs) are the most potent bronchoconstrictors known that have a crucial role in both immediate and late asthmatic responses. Montelukast effectively inhibited the activities of CysLT1Rs which is recognised in international guidelines as a novel therapy in asthma treatment [75]. According to the American College of Chest Physicians (ACCP) Evidence-Based Clinical Practice Guidelines “For patients with asthmatic cough that is refractory to treatment with ICSs and bronchodilators, in whom poor compliance or another contributing condition has been excluded, a receptor antagonist may be added to the therapeutic regimen before the escalation of therapy to systemic corticosteroids” [72]. However, there are limited clinical studies in literature that show this anti-inflammatory antagonist is an effective therapy for chronic cough.

## LEUKOTRIENES PATHWAY

The Cysteinyl leukotrienes (cysLTs) are a family of inflammatory lipid mediators including leukotriene C4 (LTC4), leukotriene D4 (LTD4) and leukotriene E4 (LTE4). Leukotrienes are produced from nuclear membrane phospholipids in multiple enzymatic cascade. They are synthesised from arachidonic acid through a number of pathways, one of which is the 5-lipoxygenase pathway [76]. In this process 5-lipoxygenase oxidated arachidonic acid to 5-hydroperoxyeicosatetraenoic acid (HpETE) and then HpETE converted to LTA4. LTA4 is an unstable leukotriene which can hydrolyse to LTC4. Alternatively, LTA4 can reform to LTB4 in neutrophils and other inflammatory cells. LTB4 is known as a strong neutrophil activator and chemoattractant. LTB4 also can cause eosinophil chemotaxis. Once LTA4 converted to LTC4, LTC4 is transported to the extracellular space where varied to LTD4 and LTE4. LTC4, LTD4, and LTE4 all have a cysteine residue and have a very similar effects on the airway smooth muscle. The cysteinyl leukotrienes are abundantly generated in the airway mucosa and submucosa by a selection of cells, mostly mast cells, eosinophils, basophils and macrophages. Cysteinyl leukotriene receptors identify CysLTs and interact with these pro-inflammatory mediators [77]. As a result of this interaction, CysLTs activate and successively recruit and stimulate inflammatory cells, increase vascular permeability, mucous secretion and bronchial hyperresponsiveness, and promote airway remodelling [78] Fig. (**2**).

## LEUKOTRIENE – ILC2 PATHWAY

A crucial question that remains unanswered is that through which mechanism(s) cysteinyl leukotriene receptors antagonists accomplish their antitussive effect on airway inflammation. Previously, it has been shown that CysLT1R is upregulated by Th2 cytokines, including IL-4 and IL-13 on human subjects [79]. Furthermore, human Th2 cells stimulated by CysLTs induced production of IL13 in CysLT1R dependant manner. LTE4 was particularly potent in inducing cytokines production for human Th2 cells compared with LTD4 [80]. Collectively, based on these reports it has been suggested that Th2 cytokines and CysLTs are synergistically regulated [81]. In a recent study in mice it was evident that ILC2 also can be stimulated by CysLTs. This study demonstrated three important findings. Firstly, it has been reported that lung and bone marrow ILC2s express CysLT1R in unchallenged mice and moreover, remains stably expressed in lung ILC2s after allergen challenges independent of STAT6 and adaptive immune cells. Secondly, stimulation of ILC2 with LTD4 (the main ligand for CysLT1R) after a single exposure to allergens, enhanced cytokine production of IL-4, IL-5 and IL-13 in CysLT1R dependant manner. Finally, it has been shown that administrating LTD4 to airways of mice regulate ILC2 to produce IL-5 and potentially cause airway eosinophilic inflammation independent of adaptive immune cells [81]. These findings are relevant in Th2 dependant diseases such as allergic and non-allergic asthma. Based on documented evidence in the above studies it can be concluded that in presence of allergens Th2 cells produce Th2 cytokines. These cytokines upregulate CysLTs in airway consequently enhance level of CysLTs which is able to active lung ILC2. Then activated ILC2s are rapidly produce Th2 cytokines in a CysLT1R dependant manner. In view of that, leukotriene - ILC2 pathway in respiratory diseases (which level of CysLTs increase) might promote airway eosinophilia inflammation and hyper responsiveness by enhancing production of IL4, IL5 and IL13 [81] Fig. (**2**).

## CLINICAL STUDIES

There are numerous studies that confirm a significant effect of montelukast on eosinophilic inflammation in asthmatic patients.

Gagro and colleagues (2004) in a study in 14 children with allergic asthma reported that after 6 weeks treatment with montelukast peripheral blood eosinophil count decreased significantly [75]. A significant decrease in the percentage of T lymphocytes and the level of total IgE was observed as well. This study monitored changes in forced expiratory volume in 1 s (FEV1) and peak expiratory flow rate (PEFR) which both improved after treatment. In a double-blind, randomized, parallel group, placebo-controlled study among 2791 adults with active seasonal allergic rhinitis treatment with montelukast 10 mg (n=813), revealed a significant reduction in peripheral blood eosinophilia in comparison with loratadine l0 mg (n=1275) and placebo (n=703) groups [82]. Similar results has been reported when airway tissue inflammatory cells were assessed directly by bronchoscopy. After 6 weeks treatment with montelukast the number of eosinophils cells and mast cell reduced significantly compared with placebo group [83].

There are several clinical studies that have reported the effectiveness of montelukast on patients with cough variant asthma (CVA) as defined by cough with bronchial hyper responsiveness and eosinophilia airway inflammation, in absence of bronchoconstriction or airway obstruction. In a study on adults, patients with chronic cough received diagnosis of CVA and AC (atopic cough).

 Two weeks therapy with montelukast demonstrated a significant decrease in cough scores which was assessed with a subjective cough symptom scale. However, in subjects with AC compared with a placebo group, cough scores did not show a significant change, so montelukast was ineffective in these patients [84]. In a small, randomized, double blind, placebo controlled trial four weeks montelukast therapy in patients with CVA demonstrated a significant improvement in cough frequency that was evaluated subjectively [85]. In another study in subjects with CVA montelukast significantly decreased the value of FeNO and sputum eosinophil. Moreover, it reduced airway hyperresponsivness and cough [86].

Two small studies have evaluated the effects of montelukast in patients with chronic cough of diverse aetiology. In a real life observational pilot study 14 patients with chronic cough (which is not due to asthma) were assessed before and after two weeks treatment with montelukast. Cough scores were measured with a validated questionnaire (Leicester Cough Questionnaire) and demonstrated a significant reduction of cough after treatment. Cough reflex sensitivity to capsaicin decreased significantly.

 While there was a decrease in cough reflex sensitivity for citric acid, it was not significant. Moreover, it has been reported that the eosinophil cationic protein (ECP) value as a marker of eosinophil activation significantly decreased [87]. In an observational study on children (n = 22) with chronic cough, four weeks treatment with montelukast was administered. In 14 children (68%) cough frequency improved within 72 hours of therapy and cough ceased by the third week of treatment.

 Children who responded to the therapy had a higher level of ECP in their pre-treatment sample compared with children who did not respond to the therapy. Absolute peripheral eosinophil blood counts and IgE levels also were significantly higher in the responders to therapy before treatment. Two of the children who did not respond to montelukast were diagnosed to have GORD. However there are no randomized controlled trials in this patient population.

## CONCLUSION

In conclusion the role of induced sputum, FeNO and blood eosinophilic to diagnose eosinophilic inflammation and monitor therapies in chronic cough has yet to be fully defined. Currently, all three methods are in use, with anti-inflammatory therapy directed at eosinophilic inflammation prescribed mainly on clinical judgement rather than evidence. There is an urgent need for the rational application of objective measures of eosinophilic inflammation to avoid excessive and potentially harmful anti-inflammatory treatment in patients with chronic cough.

## CONSENT FOR PUBLICATION

Not applicable.

## CONFLICT OF INTEREST

The author (editor) declares no conflict of interest, financial or otherwise.

## ACKNOWLEDGEMENT

Declared None.

## Figures and Tables

**Fig. (1):**
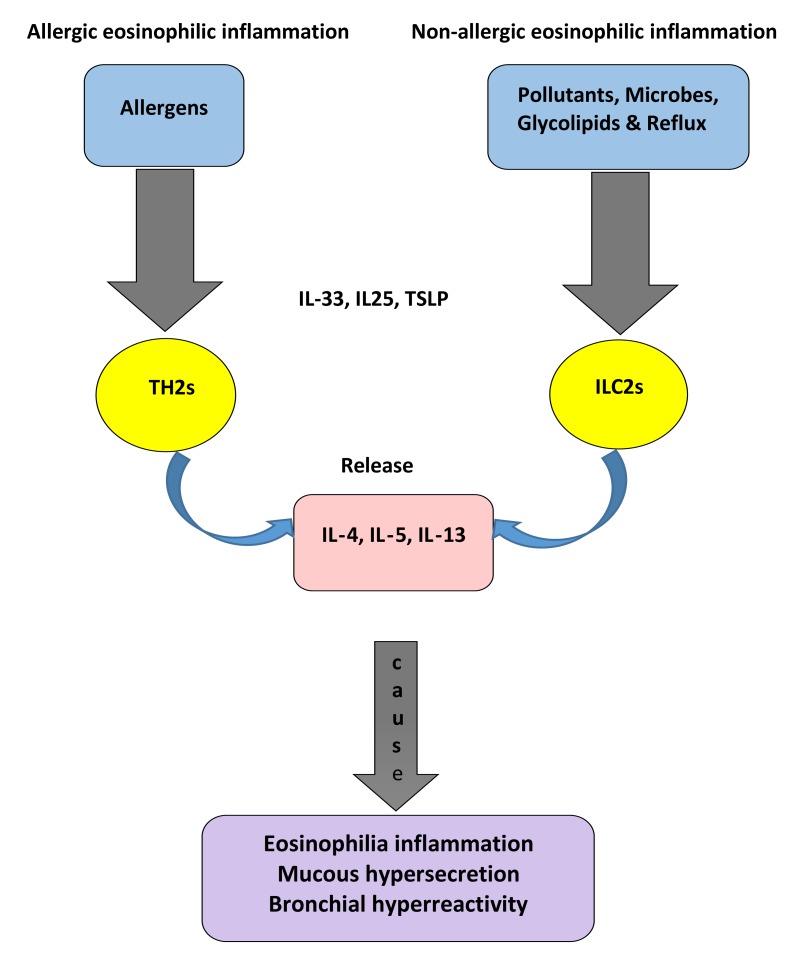
Overview of hypothetical functions of T helper type 2 (TH2) cells and innate lymphoid type 2 (ILC2) cells that lead to eosinophilic airway inflammation through two different pathways. In allergic eosinophilic airway inflammation, TH2 cells stimulated by dendritic cells in present of allergens. TH2 release interleukin-4 (IL-4), IL-5 and IL-13, and leading to immunoglobulin E (IgE) synthesis, eosinophilia inflammation and bronchial hyperreactivity. In non-allergic eosinophilic airway inflammation, ILC2s activated in present of air pollutants, microbes, glycopipids & reflux through an antigen-independent manner. Activated ILC2s release IL4, IL-5 and IL-13, causing eosinophilia inflammation, mucous hypersecretion and bronchial hyperreactivity. TH2 cells and ILC2 cells both activated in the present of IL-33, IL-25 and TSLP (thymic stromal lymphopoietin).

**Fig. (2):**
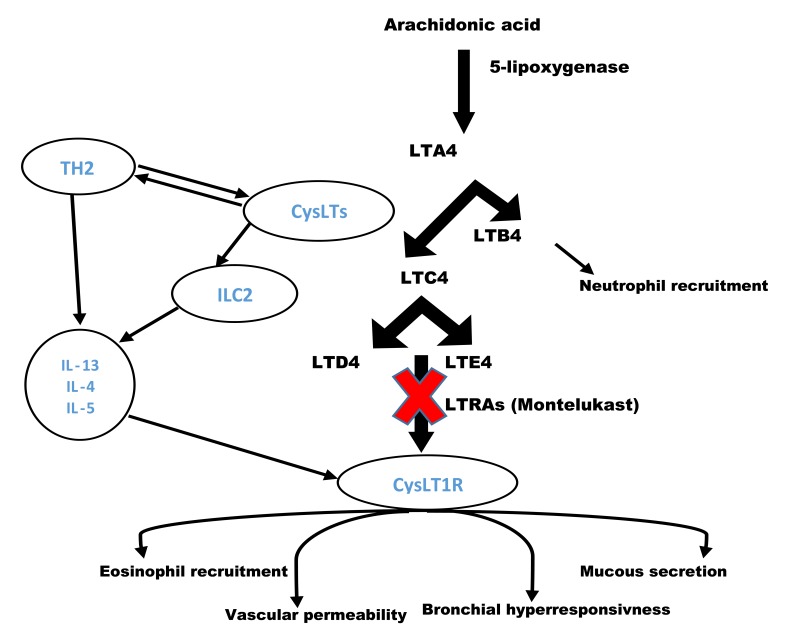
Effect of cysteinyl leukrotiens (CysLTs) on airway eosinophilic inflammation through TH2 and ILC2 pathways.
